# Paediatric Trauma Score as a non-imaging tool for predicting intracranial haemorrhage in patients with traumatic brain injury

**DOI:** 10.1038/s41598-021-00419-y

**Published:** 2021-10-22

**Authors:** Heoung Jin Kim, Sohyun Eun, Seo Hee Yoon, Moon Kyu Kim, Hyun Soo Chung, Chungmo Koo

**Affiliations:** 1 Yonsei University College of Medicine, 50-1, Yonsei-ro, Seodaemun-gu, Seoul, 03722 Republic of Korea; 2 Division of Pediatric Emergency Medicine, Department of Pediatrics, Severance Children’s Hospital, Yonsei University College of Medicine, 50-1, Yonsei-ro, Seodaemun-gu, Seoul, 03722 Republic of Korea; 3 Department of Emergency Medicine, Severance Hospital, 50-1, Yonsei-ro, Seodaemun-gu, Seoul, Republic of Korea

**Keywords:** Paediatric research, Brain injuries, Trauma

## Abstract

To identify a useful non-imaging tool to screen paediatric patients with traumatic brain injury for intracranial haemorrhage (ICH). We retrospectively analysed patients aged < 15 years who visited the emergency department with head trauma between January 2015 and September 2020. We divided patients into two groups (ICH and non-ICH) and compared their demographic and clinical factors. Among 85 patients, 21 and 64 were in the ICH and non-ICH groups, respectively. Age (p = 0.002), Pediatric trauma score (PTS; p < 0.001), seizure (p = 0.042), and fracture (p < 0.001) differed significantly between the two groups. Factors differing significantly between the groups were as follows: age (odds ratio, 0.84, p = 0.004), seizure (4.83, p = 0.013), PTS (0.15, p < 0.001), and fracture (69.3, p < 0.001). Factors with meaningful cut-off values were age (cut-off [sensitivity, specificity], 6.5 [0.688, 0.714], p = 0.003) and PTS [10.5 (0.906, 0.81), p < 0.001]. Based on the previously known value for critical injury (≤ 8 points) and the cut-off value of the PTS identified in this study (≤ 10 points), we divided patients into low-risk, medium-risk, and high-risk groups; their probabilities of ICH (95% confidence intervals) were 0.16–12.74%, 35.86–89.14%, and 100%, respectively. PTS was the only factor that differed significantly between mild and severe ICH cases (p = 0.012). PTS is a useful screening tool with a high predictability for ICH and can help reduce radiation exposure when used to screen patient groups before performing imaging studies**.**

## Introduction

### Background

Head trauma is a common injury in children. In the United States, more than 750,000 paediatric patients, i.e., approximately 150 to 400 per 100,000 people, visit the emergency department each year with a head trauma^1,2^. In Korea, patients with head trauma constitute approximately 1.8% of the total paediatric patients who visit the emergency department^3^.

Traumatic brain injury (TBI) is the most common cause of central nervous system injuries such as bruising, bleeding, and diffuse axonal damage^4^. In the United States, approximately 10% of paediatric patients who visit the emergency department with TBI require inpatient or intensive care unit (ICU) treatment, and TBI is also the most common cause of death among children and young adults^5,6^. In addition to the short-term morbidity, children with TBI may have long-term morbidities related to development such as difficulties in learning, emotional awareness, and social functioning^5,7^.

In general, mild TBI is defined as the occurrence of brain injury due to external physical vector and a Glasgow Coma Scale (GCS) score of 13–15^8,9^. Patients with mild TBI account for 75–85% of all patients with head trauma^2^. After mild TBI, changes in brain physiology can be complicated by injury such as intracranial haemorrhage (ICH)^8^. It has been reported that ICH is found in 7.5% of children with mild TBI^10^. As such, the possibility of ICH in patients with mild TBI cannot be excluded. In contrast, patients with severe TBI are defined as those with a GCS score of 8 or less^11^; these patients often require ICU hospitalization and/or neurological intervention such as placement of an extra-ventricular drain to relieve ICH-induced high intracranial pressure.

### Importance

Unlike in the adult population, imaging studies in paediatric patients are limited due to problems such as difficulty of sedation or concerns regarding exposure to radiation^12^. Radiation exposure in children is known to be associated with an increased risk of cancer and mortality, and the effect of such exposure is greater in these patients than in adults^13^. According to the Pediatric Emergency Care Applied Research Network (PECARN), age younger than 2 years, vomiting, loss of consciousness, severe mechanism of injury, severe or worsening headache, amnesia, non-frontal scalp hematoma, a GCS score less than 15, and clinical suspicion for skull fracture are suggested criteria for obtaining head computed tomography images^14^. Therefore, there is no disagreement on whether to perform imaging studies in patients with severe TBI, but this may not be the case in those with mild TBI. The PECARN criteria have high sensitivity but low specificity (sensitivity; 100%, specificity; 53.8%)^15^ and therefore, it cannot be used to effectively identify patients who do not need imaging studies. Therefore, in order to reduce unnecessary radiation exposure and sedation in paediatric patients, there is a need for predictive screening tools that can indicate the feasibility of performing imaging examinations in paediatric patients.

### Goals of this investigation

We aimed to identify a useful screening tool for paediatric patients suspected to have ICH and require imaging studies in the emergency department. Pediatric Trauma Score (PTS) is a tool that has been developed to evaluate the injury severity caused by trauma, based on weight, airway status, systolic blood pressure, level of consciousness (LOC), fractures, and wounds^16^. There is a relationship between PTS and the criteria for performing imaging studies in patients with TBI. Therefore, we investigated whether PTS could predict ICH.

## Methods

### Study design and setting

We conducted this retrospective case–control study in paediatric patients who visited Severance hospital—a tertiary care hospital in Seoul, Korea—between January 2015 and September 2020 with a diagnosis of cerebral concussion, head trauma, or TBI.

### Selection of participants

We enrolled paediatric patients under 15 years old who visited the paediatric emergency department of a tertiary care hospital due to cerebral concussion, head trauma, or TBI. Patients with severe injury underwent imaging studies according to the hospital’s protocol for head trauma, which is based on the PECARN criteria^14^. The decision to perform brain magnetic resonance imaging (MRI) was made when two or more of the PECARN criteria were satisfied. The exclusion criteria were as follows: no brain MRI at diagnosis, a medical history of cerebrovascular disease, past ICH, and a central nervous system tumour.

### Measurements

We collected medical information from electronic medical records of paediatric patients who visited the emergency department with a diagnosis of cerebral concussion, head trauma, or TBI by an emergency medical doctor. Demographic data included age and sex, and all patients were Korean. Clinical data included seizure, LOC, neurological symptoms, multiple trauma, PTS, fracture, trauma type, and ICH confirmed by imaging studies.

Seizures were classified into three types: generalized, focal, and unclear (when witness statements were inaccurate or absent). Neurological symptoms were defined as the presence of seizure, loss of consciousness, mental change, and focal neurological deficit. Trauma was classified into seven types: sports, traffic accident (TA; out-car), TA (in-car), fall down, slip down, assault, and unknown. Trauma other than head trauma was defined as the presence of trauma in other parts of the body in addition to head injury. In the PTS, each item (weight, airway, systolic blood pressure, LOC, fractures, and wounds) was assigned a score of − 1, + 1, or + 2 (Table 1), and the final score ranged from − 6 points to + 12 points^16^. ICH was classified into five types: epidural haemorrhage (EDH), subdural haemorrhage (SDH), subarachnoid haemorrhage (SAH), microbleeding, and intraventricular haemorrhage (IVH); other cases were classified as non-ICH.

**Table 1:** Paediatric Trauma Score.

Item	Score		
	+ 2	+ 1	− 1
Weight (kg)	> 20	10 to 20	< 10
Airway	Patent	Maintainable	Unmaintainable
Systolic blood pressure (mmHg)	> 90	50 to 90	< 50
Level of consciousness	Awake	Loss of consciousness	Comatose
Fractures	None	Closed or suspected	Multiple
Wounds	None	Minor	Major, penetrating, burns

The GCS was not determined in all patients. Therefore, severe cases were defined as those who required ICU hospitalization and/or neurological intervention due to ICH.

### Measures

The primary outcomes were factors affecting ICH (i.e., factors with significant differences between the ICH group and the non-ICH group). We hypothesized that PTS could be one of them. Therefore, we further analysed whether PTS could be a useful screening tool. According to the PECARN criteria, only one factor each is used as the criterion to identify paediatric patients with TBI who would require imaging. Including multiple factors such as clinical indicators could help make better decisions in the emergency department. We thought that one such potentially useful clinical indicator is PTS because PTS is an indicator that reflects the overall severity of injury, and some of the items constituting it overlap with those of the PECARN criteria.

### Analysis

We divided patients into two groups (the ICH group and the non-ICH group) and compared demographic and clinical data between the two groups using the unpaired t-test. Odds ratios (ORs) with 95% confidence intervals (95% CIs) were calculated for the significant factors. Next, cut-off values, sensitivity, specificity, and area under the curve (AUC) were calculated based on the receiver operating characteristic (ROC) curve.

Subsequently, we divided PTS into three groups based on previously known criteria (according to Tepas et al.^16^ mortality and morbidity increase significantly below 8 points) and the cut-off value calculated based on the ROC curve. The differences in ICH risk by PTS group were analysed using one-way analysis of variance (ANOVA).

Statistical analysis was performed using SPSS statistics (version 25.0; IBM Corp., Armonk, NY). A value of p < 0.05 was considered to be statistically significant.

### Ethics approval and consent to participate

This study was performed in line with the principles of the Declaration of Helsinki. Approval was granted by the Severance Hospital Ethical Committee (Severance Hospital IRB number: 2020-3356-001). This study was approved by the Severance hospital ethical committee's institutional review board for a written informed consent waiver.

### Consent for publication

All authors agree to publication.

## Results

### Characteristics of study subjects

**Figure 1:**
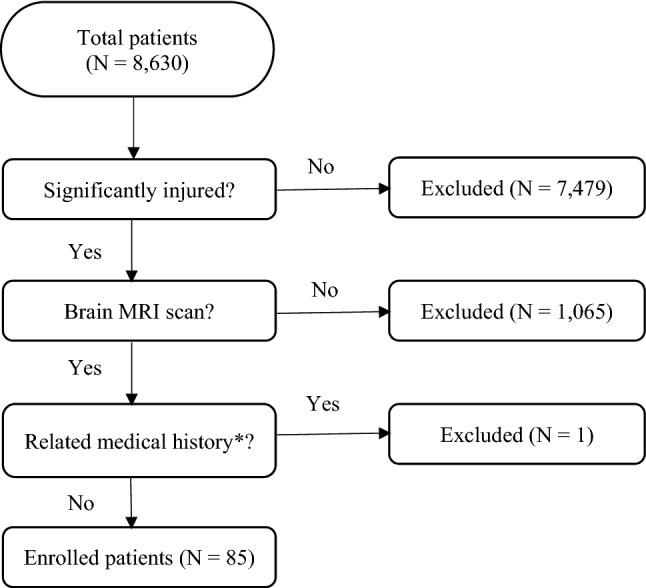
Flow chart of inclusion and exclusion criteria. *Related medical history: cerebrovascular disease, past intracranial haemorrhage, and a central nervous system tumour.

Of the total 8,630 patients, 1151 patients required imaging examinations according to the PECARN algorithm. Of those, 86 patients underwent MRI. One patient was excluded due to a history of cerebrovascular disease (a case of Moyamoya disease). Finally, 85 patients were enrolled in the study (Fig. 1).

Of the 85 enrolled patients, 21 had ICH (ICH group) and 64 did not have ICH (non-ICH group).

### Main results

The ICH group and the non-ICH group were compared in terms of age, seizure, LOC, neurological symptoms, multiple trauma, PTS, fracture, and trauma type (Table 2). Age (mean ± SD; ICH vs. non-ICH, 8.20 ± 4.483 years vs. 4.57 ± 4.864 years, p = 0.002), seizure (N [%]; ICH vs. non-ICH, 6 [9.4%] vs. 7 [33.3%], p = 0.042), PTS (mean ± SD; ICH vs. non-ICH, 11.34 ± 0.695 vs. 9.10 ± 1.700, p < 0.001), and fracture (N [%]; ICH vs. non-ICH, 1 [1.6%] vs. 11 [52.4%], p < 0.001) were significantly different between the two groups, indicating that patients in the ICH group were younger, had lower PTS, and had more seizures and fractures than those in the non-ICH group.

**Table 2:** Patient characteristics.

Total patients (N = 85)	Intracranial haemorrhage		p-value
	No (N = 64)	Yes (N = 21)	
Age (mean ± SD)	8.20 ± 4.483	4.57 ± 4.864	0.002
**Sex, N (%)**			
Male: female	47 (73.4): 17 (26.6)	12 (42.9): 9 (57.1)	0.198
**Seizure, N (%)**			
Total	6 (9.4)	7 (33.3)	0.042
Type			
Generalized	3 (50)	2 (28.6)	
Focal	1 (16.7)	0 (0)	
Unclear	2 (33.3)	5 (71.4)	
Loss of consciousness, N (%)	13 (20.3)	5 (23.8)	0.737
Neurological symptoms, N (%)	27 (42.2)	14 (66.7)	0.052
Multiple trauma, N (%)	18 (28.1)	4 (19)	0.416
Pediatric Trauma Score (mean ± SD)	11.34 ± 0.695	9.10 ± 1.700	< 0.001
Fracture, N (%)	1 (1.6)	11 (52.4)	0.001
**Trauma type, N (%)**			
Sports	3 (4.7)	2 (9.5)	0.137
TA (out-car)	10 (15.6)	1 (4.8)	
TA (in-car)	3 (4.7)	0 (0)	
Fall down	13 (20.3)	13 (61.9)	
Slip down	21 (32.8)	4 (19)	
Assaulted	10 (15.6)	0 (0)	
Unknown	4 (6.3)	1 (4.8)	

*TA* traffic accident.

Among the 21 patients with ICH confirmed by CT and MRI, 1 (4.8%) had EDH, 10 (47.6%) had SDH, 2 (9.5%) had SAH, 6 (28.6%) had microbleeding, and 2 (9.5%) had IVH. In 8 cases, ICH was not detected on CT but was detected on MRI. However, there was no case where ICH was detected on CT, but not on MRI. Of the 8 patients in whom haemorrhage was confirmed only by MRI, 4 patients had SDH and 4 had microbleeding. No patient had an underlying cause of secondary haemorrhage.

ORs were calculated for the following factors that differed significantly between the ICH group and the non-ICH group: age (OR = 0.84 [0.751–0.947, 95% CI], p = 0.004), seizure (OR = 4.83 [1.403–16.649, 95% CI], p = 0.013), PTS (OR = 0.15 [0.062–0.367, 95% CI], p < 0.001), and fracture (OR = 69.3 [8.047–596.773, 95% CI], p < 0.001; Table 3).

**Table 3:** Odds ratios of risk factors for intracranial haemorrhage.

Factor	Odds ratio	95% confidence interval	p-value
Age	0.84	0.751–0.947	0.004
Seizure	4.83	1.403–16.649	0.013
Pediatric Traumatic Score	0.15	0.062–0.367	< 0.001
Fracture	69.3	8.047–596.773	< 0.001

**Figure 2:**
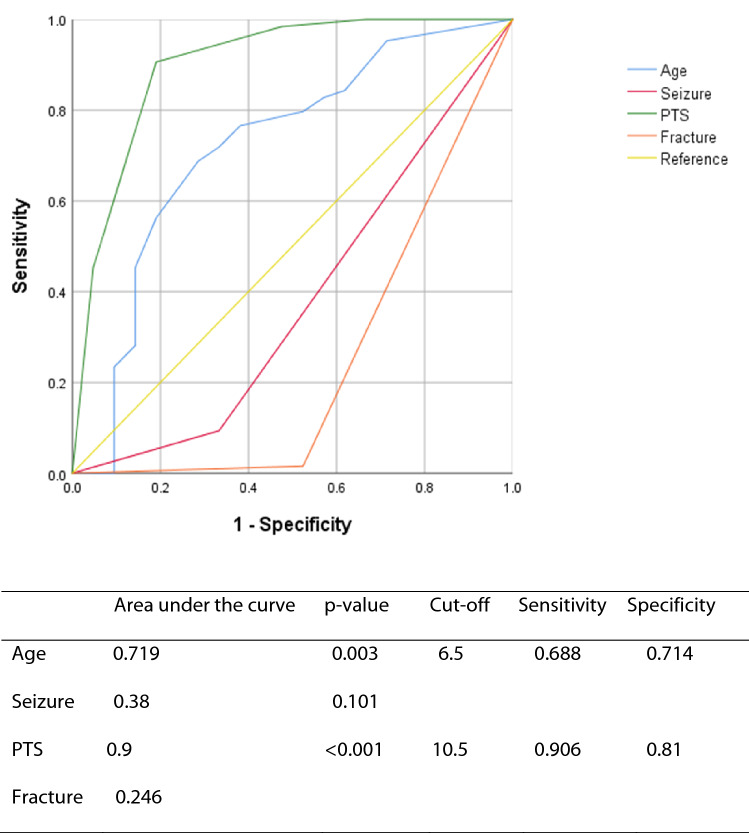
Cut-off values of risk factors for intracranial haemorrhage calculated using the receiver operating characteristic curve.

To verify whether these factors could predict ICH, we calculated their cut-off values based on the ROC curve. The factors with an AUC of 0.5 or higher were age (cut-off [sensitivity, specificity], 6.5 [0.688, 0.714], p = 0.003) and PTS (10.5 [0.906, 0.81], p < 0.001; Fig. 2).

Patients were classified into three groups based on the following criteria. Patients with a PTS score of 8 or less, which were identified as critically injured through previous studies, were classified as the high-risk group (≤ 8 points)^16,17^. In patients with a PTS exceeded 8 points, it was classified into the medium-risk group (9–10 points) and the low-risk group (≥ 11 points) based on the cut-off value of 10.5 points obtained above. The number (%) of patients in the low-risk, medium-risk, and high-risk groups was 62 (72.9%), 16 (18.8%), and 7 (8.2%), respectively. The 95% CIs for the probability of ICH in the low-risk, medium-risk, and high-risk groups were 0.16–12.74%, 35.86–89.14%, and 100%, respectively (Table 4), with significant differences between the groups as assessed using one-way ANOVA (low-risk vs. medium-risk, p = 0.001; low-risk vs. high-risk, p < 0.001; and medium-risk vs. high-risk, p = 0.026).

**Table 4:** Difference in intracranial haemorrhage risk by Pediatric Trauma Score group.

Total patients (N = 85)	N (%)	Probability (%) (95% confidence interval)	p-value	f-value
Low risk (≥ 11)	62 (72.9%)	0.16–12.74%	< 0.001	45.531
Medium risk (9–10)	16 (18.8%)	35.86–89.14%		
High risk (≤ 8)	7 (8.2%)	100%		

Of the 21 patients with ICH, four were considered severe cases as they required ICU care or neurological intervention, and two of them required extra-ventricular drain. Severe and non-severe cases were compared in terms of age, seizure, PTS, and fracture. The only factor that showed a significant difference between the two groups was PTS (p = 0.012; Table 5).

**Table 5:** Risk factor for severe intracranial haemorrhage.

Factor	Intracranial haemorrhage (N = 21)		p-value
	Non-severe (N = 17)	Severe (N = 4)	
Age (mean ± SD)	7.45 ± 4.834	5.00 ± 4.301	0.272
Seizure, N (%)	12 (70.6)	2 (50)	0.457
	5 (29.4)	2 (50)	
Pediatric Trauma Score (mean ± SD)	9.53 ± 1.419	7.25 ± 1.708	0.012
Fracture, N (%)	8 (47.1)	2 (50)	0.807
	9 (52.9)	2 (50)	

Of the 85 patients enrolled in the study, 11 (12.9%) and 26 patients (30.6%) required sedation for CT and MRI, respectively. All the patients who required sedation during CT required sedation during MRI. The average age of patients who required sedation on CT and MRI was 1.7 and 1.8 years, respectively; the oldest patient who required sedation was 6 years old overall.

### Limitations

First, this retrospective, single-centre study included too small a number of patients to demonstrate the association between PTS and ICH. Probably due to the small number of patients, we observed no cases of ICH due to assault, which is a common cause of ICH in young children^18^. Second, because of the retrospective progress of this study, the analysis related to the GCS score could not be performed due to the patients missing the GCS score; therefore, we were unable to investigate the relationship between PTS, ICH, and GCS. Third, patients who underwent brain MRI were selected, and biased results may have been derived due to sample selection bias. Therefore, larger, prospective, multicentre studies are needed in the future. In addition, it is necessary to determine the GCS in all patients with TBI to determine the association between PTS, ICH, and GCS.

## Discussion

In this retrospective study of paediatric patients with TBI, we identified young age, fracture, and seizure as risk factors for ICH requiring imaging study, as reported previously^10,14,19,20^. However, there is controversy as to whether the above-mentioned factors can be used to predict ICH. According to prospective cohort study by Dietrich et al., of 322 paediatric patients in the United States, clinical symptoms or indicators other than the GCS are not related to intracranial injury^21^. According to Haydel et al., of 175 paediatric patients who visited the emergency room with minor head injury in the United States, approximately 30–50% of patients with intracranial injury may not have a fracture, and thus, it cannot be used as a useful risk factor^22^. Moreover, Rosen et al. showed in a retrospective study of brain CT of 85 mTBI patients in the United States that there is no significant association between symptoms and GCS at hospitalization and the severity of ICH. In this study, we also found that LOC and neurological symptoms were not significantly associated with ICH^23^.

According to Borgialli et al., the GCS is a useful tool to screen clinically important TBI. This is equally valid for the paediatric GCS, which can be used in preverbal children under 2 years of age^24^. However, patients with clinically important TBI are defined as severe cases such as deaths due to TBI or patients undergoing neurosurgical intervention, and the GCS score of mild TBI is generally defined as 13–15 points^8,9^, the difference in GCS between patients with or without clinically important TBI is less than 13 points. Therefore, the GCS did not fit the purpose of our study to screen for ICH in patients with mild TBI.

PTS was developed as a predictor of injury severity in the injured child. The cut-off value for PTS is equal to or less than 8 points in severe cases of injury, for which mortality due to injury increases significantly^16,17^. PTS is useful for quickly screening patients with trauma who visit the emergency room. However, since its introduction in 1987 by Tepas et al.^16^, PTS has rarely been used for a purpose other than assessment of injury severity. One study has investigated the association between PTS and the cost of treating trauma^25^.

We screened the occurrence of ICH in patients with TBI using PTS. We found an association between PTS and ICH. The mean PTS in the ICH group was significantly lower than that in the non-ICH group (9.10 ± 1.700 vs. 11.34 ± 0.695, p < 0.001), and the OR was 0.15 (95% CI = 0.062–0.367, p < 0.001). Age, seizure, and fracture were also significantly different between the ICH group and the non-ICH group. However, ROC curve analysis revealed age (AUC = 0.719, sensitivity = 0.688, specificity = 0.714, p = 0.003) and PTS (AUC = 0.9, sensitivity = 0.906, specificity = 0.81, p < 0.001) as significant factors. Nonetheless, only the cut-off value for PTS seemed to be valuable for a screening tool with appropriate sensitivity and specificity. In addition, there was a difference in ICH risk between PTS groups, with PTS being lower in the severe ICH group.

No previous studies have investigated the association between PTS and ICH risk. PTS is a simple scoring tool that is linearly associated with the Injury Severity Score^16,17^. However, it was not a widely used scoring method because there was no specific application for PTS other than for assessment of injury severity. Moreover, Inan et al. found no correlation between blunt abdominal injuries and PTS^26^.

Our study is the first to demonstrate the relationship between PTS and specific injury. Our findings suggest that ICH risk could be screened using PTS. In addition to its significance as a screening method, the correlation between severe ICH and PTS was verified. Therefore, PTS could be helpful to determine quickly whether imaging studies are needed for patients who visit a paediatric emergency room with a head trauma.

Children are more sensitive to radiation than adults, and many efforts have been made to reduce the amount of radiation applied to children through imaging studies^12,13,27^. Besides the technical factor for radiation dose reduction, what a physician can do is minimize the number of tests exposing a child to radiation. As a screening tool of ICH, PTS can help in efforts to reduce radiation exposure caused by imaging studies in children.

Moreover, when sedation is required for imaging studies in children, there is a risk of respiratory events during sedation, as well as a delay in examination time and difficulty in appeasing an irritable child due to the fasting time for sedation. Malviya et al. reported that 5% of children experienced respiratory events during sedation; in infants, this rate was 10%^28^.

In summary, we identified the association between PTS and the risk for ICH, suggested cut-off values for imaging studies in patients at high risk for ICH, and derived criteria for performing imaging studies to reduce exposure to unnecessary radiation exposure and sedation in paediatric patients with TBI.
